# Laparoscopic and left thoracoscopic Ivor-Lewis esophagectomy for Siewert type II esophagogastric junction cancer with right aortic arch: a case report

**DOI:** 10.1186/s40792-020-01071-w

**Published:** 2020-11-18

**Authors:** Motoki Murakami, Yasutaka Nakanishi, Yudai Hojo, Tatsuro Nakamura, Tsutomu Kumamoto, Yasunori Kurahashi, Yoshinori Ishida, Hisashi Shinohara

**Affiliations:** Department of Gastroenterological Surgery, Hyogo College of Medicine, 1-1 Mukogawa-cho, Nishinomiya, Hyogo 663-8501 Japan

**Keywords:** Siewert type II esophagogastric cancer, Right aortic arch, Left transthoracic Ivor-Lewis esophagectomy, Conversion surgery

## Abstract

**Background:**

Right aortic arch (RAA) is a congenital malformation detected in 0.04% of the population without heterotaxia and makes esophagectomy and mediastinal lymphadenectomy difficult. A left thoracic approach is recommended in patients with RAA, but a minimally invasive procedure has not yet been established.

**Case presentation:**

The case was a 40-year-old man with RAA and Siewert type II adenocarcinoma of the esophagogastric junction with metastases to the adrenal glands and paraaortic lymph nodes. Conversion surgery was performed when radiologic disappearance of metastatic disease was confirmed after first-line treatment consisting of 12 cycles of S-1 plus platinum-based systemic chemotherapy. Minimally invasive laparoscopic and left thoracoscopic Ivor-Lewis esophagectomy was performed in the right semi-lateral decubitus position. The esophagus was easy to see on left thoracoscopy because of the RAA. Esophagectomy with lower mediastinal lymphadenectomy and an intrathoracic esophagogastric anastomosis was performed successfully with laparoscopy and thoracoscopy without a position change. There were no surgical complications, and no residual cancer was detected in the resected specimen on pathological examination. There has been no recurrence during 21 months of follow-up.

**Conclusions:**

Laparoscopic and left thoracoscopic Ivor-Lewis esophagectomy in the right semi-lateral decubitus position is a minimally invasive, anatomically novel procedure for Siewert type II esophagogastric junction cancer in patients with RAA.

## Background

Right aortic arch (RAA) is an intrathoracic vascular anomaly first reported by Fiotratti and detected in 0.1% of all patients and in 0.04% of the population without heterotaxia [1]. RAA is congenitally formed by occlusion of the left fourth aortic arch and persistence of the right fourth aortic arch [2]. In 96% of cases with RAA, the descending aorta lies to the right of the esophagus [3]. Dislocation of the aorta arch and thoracic aorta complicates esophagectomy and mediastinal lymphadenectomy. Although patients with RAA who have esophageal cancer usually undergo left transthoracic esophagectomy, there are no established operative methods for those with esophagogastric junction (EGJ) cancer.

Here we report a case of Siewert type II EGJ cancer with RAA for whom laparoscopic and left thoracoscopic Ivor-Lewis esophagectomy (ILE) in the right semi-lateral decubitus position was effective as a minimally invasive surgery.

## Case presentation

**Fig. 1:**
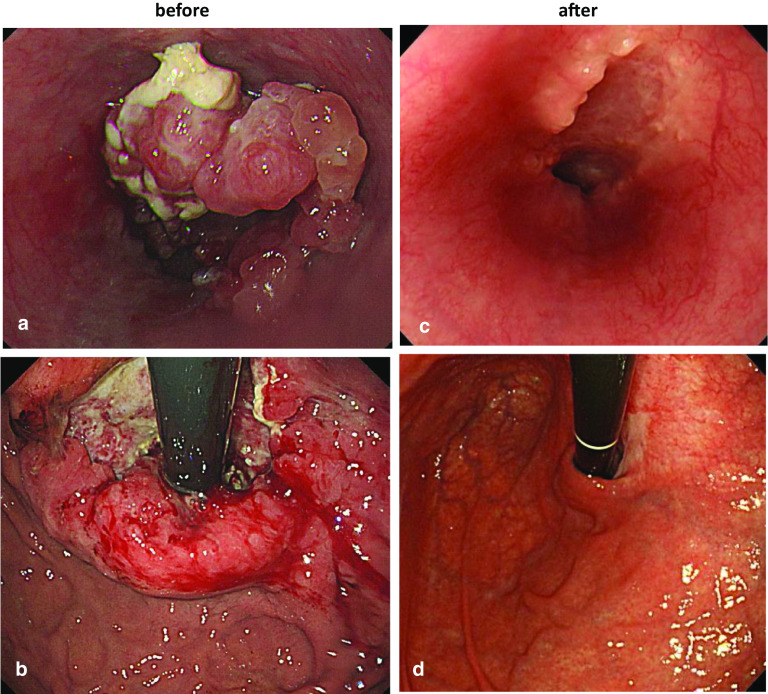
Endoscopic analyses of EGJ cancer before and after chemotherapy. **a**, **b** Upper gastrointestinal scope showed type II cancer located at the EGJ (**a**, **b**). This primary lesion was reduced after chemotherapy (**c**, **d**)

**Fig. 2:**
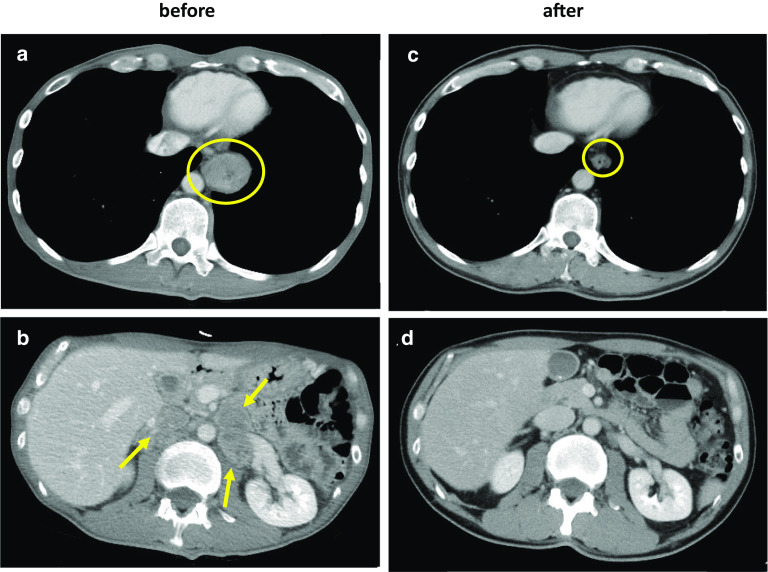
Radiological examination of the primary and metastatic sites before and after chemotherapy. There was a marked decrease in the wall thickness of the primary lesion after chemotherapy (yellow circle) (**a**, **c**). Metastatic lesions in the adrenal glands and paraaortic lymph nodes (yellow arrows) had almost disappeared after chemotherapy (**b**, **d**)

**Fig. 3:**
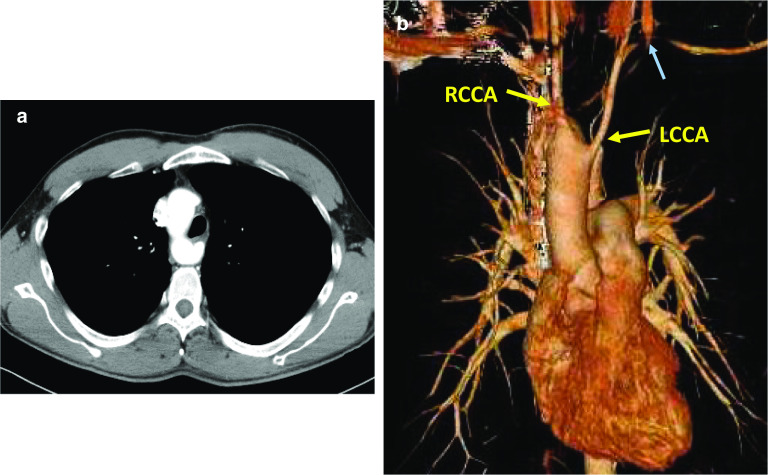
Three-dimensional CT revealed an RAA with stenosis of the left subclavian artery. Contrast-enhanced CT revealed right aortic arch (**a**). The left common carotid artery can be seen branching off the aortic arch prior to the right common carotid artery (**b**). A stenosed left subclavian artery was detected (blue arrow), and this case was classified as Edwards IIIC

A 40-year-old man with a complaint of anorexia was diagnosed with Siewert type II EGJ cancer with 8 cm of esophageal invasion (Fig. 1a, b). Histological examination revealed HER2-negative adenocarcinoma. Contrast-enhanced computed tomography (CT) revealed swelling of the paraaortic lymph nodes and both adrenal glands (Fig. 2a, b). Meanwhile, the patient was found to have an RAA malformation. On three-dimensional CT, the malformation was classified as Edward type IIIC, in which the left common carotid artery branches off the aortic arch before the right common carotid artery, and the left subclavian artery was isolated from the aortic arch (Fig. 3a, b). No cardiac anomaly was detected on echocardiography. The final diagnosis was stage IV Siewert type II EGJ cancer with distant metastases. The patient received 10 cycles of systemic chemotherapy consisting of S-1 and oxaliplatin without gastrostomy. Subsequently, oxaliplatin was changed to cisplatin when he developed grade 2 peripheral neuropathy, and 2 more cycles were added. After chemotherapy, there was a significant reduction in the primary lesion and distant metastases, and cancer cells were not detected by biopsy of the primary lesion (Figs. 1c, d and 2c, d). Furthermore, there was no significant uptake in the primary and metastatic lesions on 18-fluorodeoxyglucose positron emission tomography. To reduce the risk of disease progression in the primary lesion if it became refractory to chemotherapy, we elected to perform conversion surgery consisting of esophagectomy with lymph node dissection. Essentially, it is necessary to dissect the upper and middle mediastinal lymph nodes in Siewert type II EGJ cancer with extensive esophageal invasion. However, we anticipated that conversion surgery with dissection of both the upper and mediastinal lymph nodes would be too invasive in a patient with RAA. Therefore, the initial plan was to dissect the nodes from stations 1, 2, 3a, 4sa, 5, 6, 7, 8a, 9, 11p, 19, and 20, and the lower mediastinal nodes at stations 110, 111, 112aoA, 112pulR, and 112pulL. The adrenal glands were preserved because the metastases therein had disappeared radiologically.

**Fig. 4:**
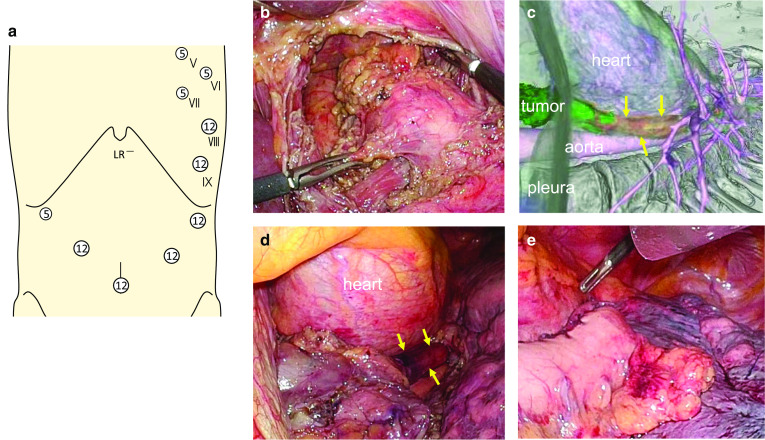
Radiological and surgical images showing left thoracoscopic ILE with an RAA. Schema for the port setting (**a**). Overview of the laparoscopic lower mediastinal lymphadenectomy performed (**b**). A trans-hiatal approach was performed without being impeded by the RAA. Radiological images obtained by three-dimensional CT reconstruction using Synapse Vincent software (Fujifilm, Tokyo, Japan) (**c**) were compared with the surgical field (**d**) under a left thoracoscopic approach. Yellow arrows indicate the esophagus. Both images show that the esophagus was located to the left of the descending aorta and would be visible with left thoracotomy. An intrathoracic esophageal–gastric anastomosis was constructed via a left transthoracic approach (**e**)

Laparoscopic and left thoracoscopic ILE was planned to avoid the right descending aorta. The patient was fixed to the operating table in the right semi-lateral decubitus position and then rotated to the supine position prior to abdominal manipulation. Five abdominal ports were used, comprising a 12-mm umbilical port for the camera, 12-mm ports in the upper abdomen on both sides, a 12-mm left subcostal port, and a 5-mm right subcostal port (Fig. 4a). A Nathanson liver retractor was inserted through the epigastric region. First, the lower mediastinal lymph node dissection (which could not be performed via a later left thoracoscopic approach due to the RAA) was performed using a trans-hiatal approach (Fig. 4b). After dissection of the celiac lymph nodes, the cardiac portion of the stomach was resected with a sufficient margin. Next, the operating table was rotated to the right lateral decubitus position. The ports placed for left thoracoscopy consisted of a posterior 12-mm port for the camera in the 11th intercostal space (ICS), an anterior 5-mm port in the 5th and 7th ICS, a posterior 5-mm port in the 6th ICS, and a posterior 12-mm port in the 8th ICS (Fig. 4a). Under left thoracoscopy, the middle and lower parts of the esophagus were easily observed, in agreement with the three-dimensional CT findings (Fig. 4c, d). The gastrointestinal conduit was pulled through the esophageal hiatus into the left thorax using a thoracoscope and a laparoscope to ensure there were no twists. An intrathoracic functional end-to-end esophagogastric anastomosis was created at the middle mediastinum (Fig. 4e). Operating time was 654 min and blood loss was minimal.

**Fig. 5:**
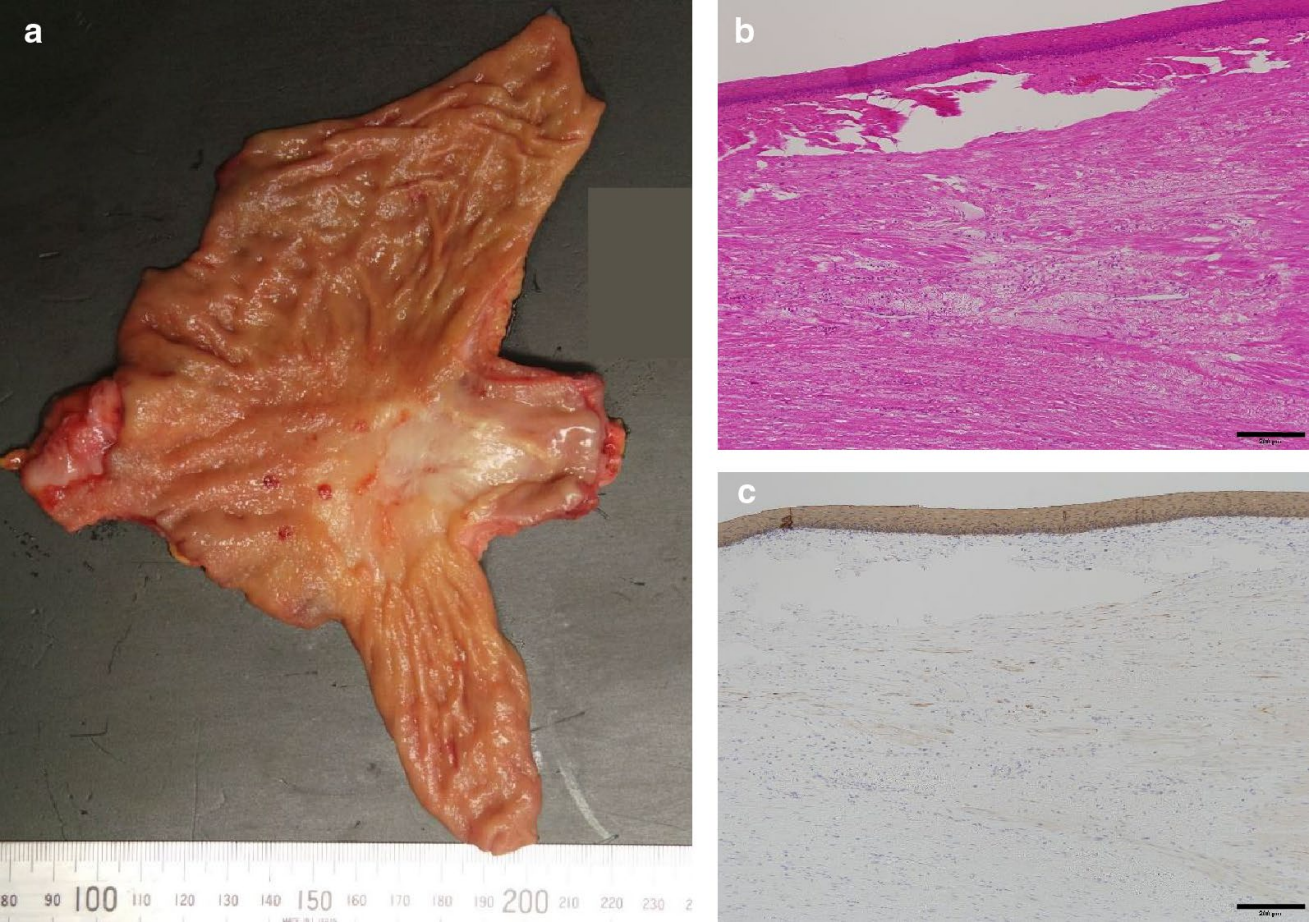
Pathological examination. Resected gastroesophageal specimen (**a**). The primary site was unclear compared with before chemotherapy. There was no residual cancer in the resected specimen, which was stained with hematoxylin and eosin (**b**) and anti-pan cytokeratin antibody (AE1/AE3) (**c**) (bar, 200 µm)

Pathological examination of the resected specimen with hematoxylin–eosin and anti-pancytokeratin antibody staining revealed no cancer (Fig. 5a–c). The postoperative course was uneventful and the patient was discharged 15 days after surgery. There were no complaints related to gastroesophageal reflux disease, and upper gastrointestinal endoscopy performed 7 months postoperatively did not reveal any reflux esophagitis. So far, he has been recurrence-free for 21 months with no adjuvant chemotherapy.

## Discussion

Several clinical studies have investigated the surgical approach for Siewert type II EGJ cancer. Upper and middle mediastinal lymph node metastases are detected more frequently in patients with more than 3 cm of esophageal invasion than in those with less invasion [4]. In Siewert type II EGJ cancer with extensive esophageal invasion, mediastinal and celiac lymph node dissection via a right thoracoabdominal approach has been recommended because there are few lymph node metastases along the greater omentum and around the pylorus [5, 6]. The ILE technique reported by Lewis in 1946 consists of middle and lower esophagectomy and proximal gastrectomy with two-field lymphadenectomy [7]. In patients with Siewert type II disease, the prognosis was reported to be better after right transthoracic ILE than after trans-hiatal extended gastrectomy with D2 and lower mediastinal lymphadenectomy [8]. ILE is usually performed via a right transthoracic approach because esophagectomy is anatomically straightforward. Furthermore, more lymph nodes can be resected with right thoracotomy than with left thoracotomy [9]. There is no evidence of a significant difference in prognosis between a trans-hiatal approach and a right or left thoracoabdominal approach in patients with less than 3 cm of esophageal invasion [10–12]. Thus, the left thoracoabdominal approach would not be expected to improve the outcome compared with a right thoracoabdominal and trans-hiatal approach in Siewert type II EGJ cancer.

The left thoracoabdominal approach was reported by Akiyama in 1979 [13]. Its advantages are that the mediastinum and upper abdomen can be observed at the same time without changing position and the operating time is shorter. However, the left thoracoabdominal approach is an invasive method for dissection of the diaphragm and thoracoabdominal wall. Moreover, it is very difficult to operate on the entire thoracic esophagus because of the location of the aortic arch. Left thoracoscopic ILE was able to offset these two disadvantages in this case. A left thoracoscopic and laparoscopic approach is minimally invasive without diaphragmatic dissection, and the approach to the esophagus is straightforward with left thoracotomy when RAA is present. A minimally invasive abdominal and left thoracic approach for EGJ cancer was reported by Takeuchi et al. [14]. This approach enabled the lower esophagus to be resected under a thoracic view with an adequate margin. Furthermore, an intrathoracic esophago-jejunal anastomosis could be performed both laparoscopically and thoracoscopically without a change in position. Compared with the trans-hiatal laparoscopic approach, laparoscopic and left thoracoscopic reconstruction affords a better surgical field and can be performed safely, especially in EGJ cancer with extensive esophageal invasion. This surgical approach is useful and feasible in patients with EGJ cancer and RAA.

We searched the literature in the PubMed and Japan Medical Abstracts Society electronic databases from 1975 to 2019 using the keywords “right aortic arch” and “esophagectomy”. Including our case, there have been 35 reports of esophagectomy in patients with RAA. The patients comprised 32 men and 3 women aged 30–78 years and included 5 cases (14.3%) with a diagnosis of adenocarcinoma. Thirty-four cases (97.1%) with RAA underwent left thoracotomy. In these reports, lymph node dissection around the right recurrent laryngeal nerve was most difficult to perform with left thoracotomy alone. In contrast, using a trans-hiatal approach, the lower mediastinal lymph nodes could be dissected in exactly the same way as in patients without RAA. There is one report of right transthoracic ILE and another of left transthoracic ILE being used to treat EGJ cancer [15, 16]. In the right transthoracic ILE case, the middle and lower esophagus were isolated from the descending aorta by aortic elongation [15]. Right transthoracic ILE would have been difficult in our patient in view of his slender habitus and lack of aortic elongation. We suggest that preoperative three-dimensional CT imaging is a useful tool for estimating the locations of the esophagus and descending aorta when planning esophagectomy in a patient with RAA. There is a report of left transthoracic ILE in a patient with previously repaired tetralogy of Fallot in whom thoracoscopic esophagectomy was performed in a right-sided position after neoadjuvant chemotherapy [16]. In our case, we performed left transthoracic ILE laparoscopically and thoracoscopically in the right semi-lateral decubitus position. The advantage of the right semi-lateral decubitus position is that the operation could be performed with bed rotation only. Avoidance of a change in position is preferable for maintenance of general anesthesia.

Generally, esophagectomy with mediastinal lymph node dissection is performed in patients with EGJ cancer and extensive esophageal invasion. In our case, there was no evidence of cancer in the primary lesion on endoscopic biopsy or any radiological evidence of residual metastatic lesions. In esophageal cancer with a clinical complete response to neoadjuvant therapy, disease-specific survival and recurrence-free survival were better after esophagectomy than after nonsurgical treatment [17]. Kurokawa et al. found that 5.1% of their patients with EGJ cancer and extensive esophageal invasion had metastasis to the lymph nodes at station 106recR (6). Generally, the station 106recR lymph nodes plus the middle and lower mediastinal lymph nodes should be dissected. However, we performed left transthoracic ILE without upper or middle mediastinal lymphadenectomy as conversion surgery because of the marked response to chemotherapy and the difficulty of performing an upper mediastinal lymph node dissection in a patient with RAA. The patient has been recurrence-free during 21 months of follow-up. This procedure might not be suitable as radical surgery in non-conversion cases or in patients with squamous cell carcinoma of the esophagus.

## Conclusions

We successfully treated a patient with Siewert type II EGJ cancer and RAA by conversion surgery after systemic chemotherapy. Laparoscopic and left thoracoscopic ILE in the right semi-lateral decubitus position is a minimally invasive and anatomically novel approach in Siewert type II EGJ cancer with RAA.

## Data Availability

Not applicable.

## Abbreviations

RAA: Right aortic arch
EGJ: Esophagogastric junction
ILE: Ivor-Lewis esophagectomy
CT: Computed tomography
ICS: Intercostal space

## References

[1] Hastreiter AR, D’Cruz IA, Cantez T, Namin EP, Licate R (1966). Right-side aorta I. Occurrence of right aortic arch in various types of congenital heart disease II. Right aortic arch, right descending aorta, and associated anomalies. Br Heart J..

[2] Stewart JR, Kincaid OW, Titus JL (1966). Right aortic arch: plain film diagnosis and significance. Am J Roentgenol Radium Ther Nucl Med.

[3] Knight L, Edwards JE (1974). Right aortic arch. Types and associated cardiac anomalies. Circulation.

[4] Kurokawa Y, Hiki N, Yoshikawa T, Kishi K, Ito Y, Ohi M (2015). Mediastinal lymph node metastasis and recurrence in adenocarcinoma of the esophagogastric junction. Surgery..

[5] Yoshikawa T, Takeuchi H, Hasegawa S, Nozaki I, Kishi K, Ito S (2016). Theoretical therapeutic impact of lymph node dissection on adenocarcinoma and squamous cell carcinoma of the esophagogastric junction. Gastric Cancer.

[6] Kurokawa Y, Takeuchi H, Doki Y, Mine S, Terashima M, Yasuda T (2019). Mapping of lymph node metastasis from esophagogastric junction tumors: a prospective nationwide multicenter study. Ann Surg.

[7] Lewis I (1946). The surgical treatment of carcinoma of the oesophagus; with special reference to a new operation for growths of the middle third. Br J Surg.

[8] Susanne B, Thomas S, Patrick H, Moritz JS, Leila S, Ulrike H (2018). Surgical strategies in true adenocarcinoma of the esophagogastric junction (AEG II): thoracoabdominal or abdominal approach?. Gastric Cancer.

[9] Duan X, Shang X, Tang P, Jiang H, Yu Z (2018). Lymph node dissection for Siewert II esophagogastric junction adenocarcinoma: a retrospective study of 136 cases. ANZ J Surg.

[10] Hulscher JB, van Sandick JW, de Boer AG, Wijnhoven BP, Tijssen JG, Fockens P (2002). Extended transthoracic resection compared with limited transhiatal resection for adenocarcinoma of the esophagus. N Engl J Med.

[11] Sasako M, Sano T, Yamamoto S, Sairenji M, Arai K, Kinoshita T (2006). Left thoracoabdominal approach versus abdominal-transhiatal approach for gastric cancer of the cardia or subcardia: a randomized controlled trial. Lancet Oncol.

[12] Kurokawa Y, Sasako M, Sano T, Yoshikawa T, Iwasaki Y, Nashimoto A (2015). Ten-year follow-up results of a randomized clinical trial comparing left thoracoabdominal and abdominal transhiatal approaches to total gastrectomy for adenocarcinoma of the oesophagogastric junction or gastric cardia. Br J Surg.

[13] Akiyama H, Miyazono H, Tsurumaru M, Hashimoto C, Kawamura T (1979). Thoracoabdominal approach for carcinoma of the cardia of the stomach. Am J Surg.

[14] Takeuchi Y, Ebihara Y, Nakanishi Y, Asano T, Noji T, Kurashima Y (2020). A minimally invasive abdominal and left thoracic approach as a palliative treatment for adenocarcinoma of the esophagogastric junction with severe stenosis: a case report. Asian J Endosc Surg.

[15] Linson J, Latzko M, Ahmed B, Awed Z (2017). Minimally invasive Ivor-Lewis esophagectomy for esophageal cancer with right aortic arch. Gastrointest Oncol.

[16] Thomas MJ, Barlett HL, Basetti MF, Lubner SJ, Kirvassilis G, Anagnostopoulos PV (2017). Minimally invasive esophagectomy in a patient with tetralogy of Fallot and right-sided aortic arch. Ann Thorac Surg.

[17] Ohkura Y, Shindoh J, Ueno M, Iizuka T, Udagawa H (2018). Comparision of outcome of esophagectomy versus nonsurgical treatment for resectable esophageal cancer with clinical complete response to neoadjuvant therapy. Ann Surg Oncol.

